# The anatomical knowledge of Namibian school children

**DOI:** 10.1002/ase.70164

**Published:** 2025-12-01

**Authors:** Adam M. Taylor, Lojandrie Kirsten, Luigi Sedda, Quenton Wessels

**Affiliations:** ^1^ Lancaster Medical School, Faculty of Health and Medicine Lancaster University Lancaster UK; ^2^ Division of Anatomy, School of Medicine University of Namibia Windhoek Namibia

**Keywords:** Africa, anatomical knowledge, engagement, learners, Namibia, school children

## Abstract

The public has limited knowledge of key organs and anatomical structures. The lack of anatomical knowledge and understanding can hinder time to access healthcare, quality of care, and treatment outcomes. The current study investigated the anatomical knowledge among Namibian children by comparing 8 school grades—4 to 12, which covers children from the ages of 9 to 18 years old, with a total of 481 participants from 5 schools located across various areas of Namibia. All structures showed an increase in the correct responses with an increase of age except for the stomach. Structures within the abdomen were most poorly answered, with the spleen (8.8%), adrenals (9.8%), gallbladder (11.9%), and pancreas (12.1%). Structures were then grouped into functional systems and a multiple logistic regression model was used to ascertain knowledge level compared with grade 4 (the earliest year of education) and girls as reference. Knowledge improved significantly across multiple increasing school grades. Students demonstrated the best increase in performance in muscular, endocrine, respiratory, digestive, and nervous systems, respectively—with 2 schools outperforming the rest. Analysis of the percentage of structures correctly located by boys and girls showed that girls outscored boys on average, in 15 of the structures. This is the first study to look specifically at the anatomical knowledge of children in both a primary and secondary educational setting, and the first that considers learners in Africa. The study provides evidence into the need for improved health education and promotion and its benefits in school children and their anatomical knowledge.

## INTRODUCTION

Children's understanding of health and disease is guided by their knowledge and understanding of their individual body parts.^1^ These body parts can undergo detrimental changes in disease and illness to present symptoms and could ultimately result in death throughout life.^2^, ^3^ Body knowledge is conceptualized as the confluence of three levels of knowledge about the human body, namely: lexical‐semantic, sensorimotor, and visuospatial (as assessed in this study).^4^ Lexical knowledge, within the current context, refers to anatomical words and vocabulary. Semantic knowledge in turn refers to the ability to combine the words in a meaningful manner.^4^, ^5^ Lexical–semantic body knowledge is at a level beyond that which would be expected from participants in the context of this study but complements sensorimotor body knowledge (knowledge of the body's relative position in space–time) and visuospatial body knowledge (based on visual cues which permit an understanding of the boundaries and topology of body parts).^4^, ^5^ An individual's visuospatial knowledge of anatomy, hereafter referred to as anatomical knowledge, refers to their knowledge of the names of body parts, their location and (potential) functions, and how these elements are interlinked.

The public's anatomical knowledge has been shown to be lacking, with recent studies in the UK and Hong Kong, demonstrating a varying but overall poor performance in knowledge of multiple basic anatomical structures and their locations.^3^, ^6^ These recent studies confirm findings from earlier years that anatomical knowledge of patients, even those suffering from specific diseases of organs, has limited knowledge of where their afflicted organs (and others) are located; individuals undergoing surgery or with pelvic organ prolapse also have limited knowledge too.^7^, ^8^, ^9^

This limited knowledge in patients can be a barrier to seeking timely and appropriate care, if unbeknownst to the sufferer what organ might be causing pain and whether the pain is referred, but equally could be a source of anxiety. For instance, if a patient is being admitted for gallbladder surgery and assumes the bladder refers to urine, they may suffer significant anxiety immediately before surgery if they were to learn that the organ is in the upper abdomen. It may be even worse if they wake up expecting to see surgical scars around the lower abdomen/pelvis but see them in the upper right quadrant. These example situations demonstrate that these issues could have been avoided with better anatomical or health literacy.^10^ Furthermore, the patient's health literacy and effective communication by the healthcare provider have an impact on informed and written consent.^11^

Cheung and colleagues showed an inverse relationship of age with level of knowledge with the peak knowledge appearing in the age 16–25‐year bracket. The data also suggest that those presenting only primary education level performed better than those with a secondary education as their highest level of attainment, but not significantly.^6^

Even with historical studies and those more recent, it is hard to see any improvement in patient or public anatomical knowledge. This is intriguing given the increasing investment in public health campaigns by governments in many countries to reduce the impact of disease(s) and try to improve lifestyle choices.

The driver of these health campaigns is often to reduce the incidence and/or disease burden of a given condition, particularly those that generate the most impact on health and social care provisions. In recent anatomical knowledge studies in the UK and Hong Kong, there were intriguing observations, particularly in the UK study. From an anatomical perspective, the fact that <2 of 3 respondents could correctly identify the location of the heart was surprising.^3^, ^6^ Given the significance of the heart in basic bodily functions and its involvement as the leading cause of death in the UK through ischemic heart disease in 2022, and similarly, the 4th highest contributor to death in Hong Kong. Over 59,000 deaths in the UK were caused by ischemic heart disease, over 10% of all deaths, and in Hong Kong, it accounted for 11.3% of male deaths and over 10.7% of female deaths in 2022.^12^, ^13^ This is of interest because there is an opportunity for basic anatomical knowledge to feed into people making decisions about their health which could improve their life expectancy or reduce their disease burden by seeking earlier help. There is limited information relating to what children know about anatomical structures in the body; greater understanding of the body and health is important in school children, Much of the learning about health can inform decisions they make in later life about activities, such as healthy eating, smoking, drinking, drug use and other things that have an impact on quality of life, morbidity, and mortality.^14^, ^15^, ^16^ There is also potential that later in life, this information may be valuable in these individuals making informed choices and understanding about their health or that of their families, improving health literacy and utilization of healthcare resources.^17^

The anatomical knowledge of participants in the current study will provide a useful insight into the knowledge of a younger population. The study will also examine how girls and boys in Namibian education, in the absence of confounders, perform relative to each other in a system where equal access to educational time and resources in sub‐Saharan Africa is often a challenge.^18^

This study of children in Namibia represents the first study of its kind examining a young population, that of a country in Africa and one which is in the upper‐middle income classification. The study setting in a sub‐Saharan African country is an important one. In these countries, access to education and completion of it, is a challenge for many women, particularly in rural and remote areas which often results in a significant gender gap, not just in performance but also in attendance.^19^ Data on performance in primary education is limited; it shows girls are better at reading, but there is little difference in maths performance.^20^ However, the situation changes at secondary and tertiary levels, where girls still face barriers. It is well recognized that in terms of southern African countries, Namibia is one of the more progressive countries working hard and achieving good progress toward gender equality as government policies such as the National Gender Policy aim to reduce gender disparities across all sectors, not just education.^21^, ^22^ It is also important to recognize that the geography of a country such as Namibia, with a significant rural and remote population, can make accessibility to an educational setting a big challenge for pupils, particularly girls, to get there safely.^23^ Many parents are reluctant to send their daughters to school, particularly secondary school due to safety concerns during these journeys in rural or remote areas.^24^, ^25^

Economic challenges also disproportionately affect girls in sub‐Saharan countries such as Namibia. Primary education across Namibia is free, but this does not include costs associated with schooling such as transportation, where available, cost of uniforms or any reading or writing materials that may be required.^26^ This often results in boys being prioritized for attending school over their sisters. This disproportionately affects girls, especially in poorer families, as they may be required to work at home providing care or helping with family existence such as farming.^27^ All of these factors combined demonstrate why education can be challenging for girls in Namibia, where 33% of them leave school before their high‐school graduation, regardless of the compulsory education required years in school.^28^ Any of these situations will result in girls having less access to educational time and the expectation that they would perform more poorly compared with their boy counterparts.

### Study setting

Namibia sits as an upper‐middle‐income country as classified by the World Bank; in the last 30 years, it has seen a substantial educational reform. This reform to the Cambridge system has increased the number of learners accessing educational provision. In contrast to its alignment as an upper‐middle‐income country, it still sees a considerable number of deaths from communicable diseases such as HIV/AIDS, Tuberculosis, and maternal deaths.^18^

Namibia is a country that invests heavily in its education, with one of the highest gross domestic product (GDP) expenditures on education in the world.^29^ Statistical data from the World Bank indicate an average of 8.6% of Namibia's GDP was allocated to education from 2010 to 2023.^30^ The majority (around 87%) of the 1964 schools within Namibia are state‐run and English is the primary medium of instruction for learners aged 9 years and above. However, the early years of tuition, for learners aged 6 to 9, can be provided in their mother tongue, such as Khoekhoegowab, Oshiwambo, German, Afrikaans, RuKwangali, Otjiherero, and other indigenous languages. Primary school education, for learners aged between 6 and 13, is phased into Junior Primary (pre‐primary to grade 3) and Senior Primary (grades 4 to 7) and is followed by so‐called high school which is phased as Junior Secondary (grades 8 to 10) and Senior Secondary (grades 11 to 13).^30^, ^31^ A core common curriculum, The National Curriculum for Basic Education, is followed throughout the primary and secondary educational settings and covers numerous anatomical structures and systems as part of Health Education (grades 4 to 7) and Life Science (grades 8 and 9). Schooling is compulsory from the age of 6 to 16 and learners aspiring to tertiary education are required to obtain an International General Certificate of Secondary Education (Cambridge Assessment International Education) during their final two years of tuition (grades 11 and 12 at ages 17 and 18).^31^ An A‐level program, which aligns with the British educational system and is considered part of the Senior Secondary phase, equates to grade 13 and is offered by some private schools.^31^ Although overall it has been shown that anatomical knowledge is lacking in the general population, this is the first study to look specifically at the anatomical knowledge of children in both a primary and secondary educational setting, as well as the first study of this type to look at a population within Africa.

## MATERIALS AND METHODS

A survey, similar to that used in previously published studies, was distributed to a number of private schools in Windhoek, Walvis Bay, and Keetmanshoop, which are the 1st, 3rd, and 13th most populous cities in Namibia in 2024.^3^ Schools in these cities were selected as they responded to requests to be involved, as well as locations that the research team was able to access safely. The survey asked participants to place a marker detailing their understanding of the location of various organs and structures within the body: adrenals, appendix, bladder, biceps, brain, cornea, cruciate ligament, diaphragm, gallbladder, hamstrings, heart, kidneys, liver, lungs, pancreas, quadriceps, spleen, stomach, thyroid, and triceps. The Achilles was used as a demonstration of the task required and reported as 100% on the results for perspective and comparison to all tested organs/structures. Demographic information relating to age, school grade, and gender was anonymously collected. The study was approved by the Namibian Ministry of Health and Social Services (reference #: LK2022).

From these 3 cities, 5 private schools took part—1 from Keetmanshoop, Keetmanshoop Private School; 2 from Walvis Bay, International School of Walvis Bay, Walvis Bay Private School; and 2 from Windhoek, Rosewood Academy and Windhoek Gymnasium. Each school offers primary and secondary education to boys and girls. These private (non‐state) schools follow recognized international curricula such as Cambridge or International Baccalaureate that are validated by the Ministry of Education, Arts and Culture. The Education Act 2001 section 16 covers private schools' curriculum following their submission and application for approval of their curriculum, which also examines the content, the resources available for delivery of the curriculum, as well as staff involved. In this study, schools followed one of the approved curricula: Cambridge International Curriculum, Namibian Senior Secondary Certificate (NSSC), Independent Examinations Board or a combined Namibian National Curriculum and Cambridge International Curriculum. Questionnaires were administered in classrooms with researchers present to confirm there was no information sharing between students or from teachers to students, as well as ensuring any health promotion material or posters were not assisting students with answers. This was needed as many of the resources in classrooms have an association with health education to reduce transmission of diseases and improve quality of life and life expectancy.^32^, ^33^, ^34^

The responses were grouped according to their school grades (4 to 12), covering children from the age of 10–18. These represent the latter 3 years of primary school and the 5 years of secondary school in the Namibian education system. These year groups/ages were chosen as they were deemed competent in reading and understanding the task as part of this research and represent ages at which an understanding of the body begins to form, as well as having teaching about various parts of the body delivered as part of the curriculum.

A total of 501 school pupils volunteered and returned completed answer sheets; 20 of these were excluded due to being incomplete. This gave a final total of 481 completed answer sheets which were then double‐blind marked against a master copy and scores were transcribed into an Excel spreadsheet for analysis. Comparison of overall girls vs. boys' performance was done by the ANOVA test.

To determine whether the pupil performance is associated with gender and grade, while controlling for the variability arising from the schools attended by the children, logistic generalized linear mixed models with binomial error structure were employed. The outcome was the number of exact answers over 20 questions (or less in the case of the performance on individual body systems), and gender and grade were the fixed effects, with girls and grade 4 as reference categories, respectively. The school ID was used as a random effect in these instances. The resulting random effect intercepts were then explored to evaluate school performance while controlling for all other variables.

A similar model was employed for the performance on each body system: endocrine (adrenals, pancreas and thyroid), digestive (appendix, gallbladder, liver and stomach), excretory (bladder and kidneys), musculoskeletal (biceps, cruciate ligament, hamstrings, quadriceps and triceps), nervous (brain and cornea), respiratory (diaphragm and lungs), circulation (heart), and immune (spleen). In this case, each grade was explored as a reference category to investigate statistically significant differences between grades and performance.

The generalized linear mixed models were fitted by maximum likelihood using the Laplace approximation. All statistical analyses were conducted in either R v4.3.2, using the lme4 package and Graphpad Prism 5.^35^

The sample size for each school and the breakdown of gender of respondents are shown in Table 1.

**TABLE 1:** Demographic data of each school's respondents by grade and gender.

	School 1			School 2			School 3			School 4			School 5		
	Total pupils	Boys	Girls	Total pupils	Boys	Girls	Total pupils	Boys	Girls	Total pupils	Boys	Girls	Total pupils	Boys	Girls
Grade 4	6	3	3	3	3	0	10	1	9	10	0	10	26	11	15
Grade 5	8	6	2	15	7	8	9	3	6	10	3	7	21	9	12
Grade 6	7	4	3	16	10	6	18	6	12	10	4	6	32	15	17
Grade 7	0	0	0	15	8	7	10	4	6	9	4	5	13	4	9
Grade 8	5	5	0	15	5	10	10	2	8	10	5	5	13	6	7
Grade 9	8	3	5	15	7	8	10	2	8	5	0	5	12	9	3
Grade 10	6	3	3	11	4	7	10	4	6	11	3	8	28	10	18
Grade 11	10	4	6	11	3	8	11	2	9	8	3	5	0	0	0
Grade 12	0	0	0	9	6	3	10	6	4	5	2	3	0	0	0
Total	50	28	22	110	53	57	98	30	68	78	24	54	145	64	81

## RESULTS

**FIGURE 1:**
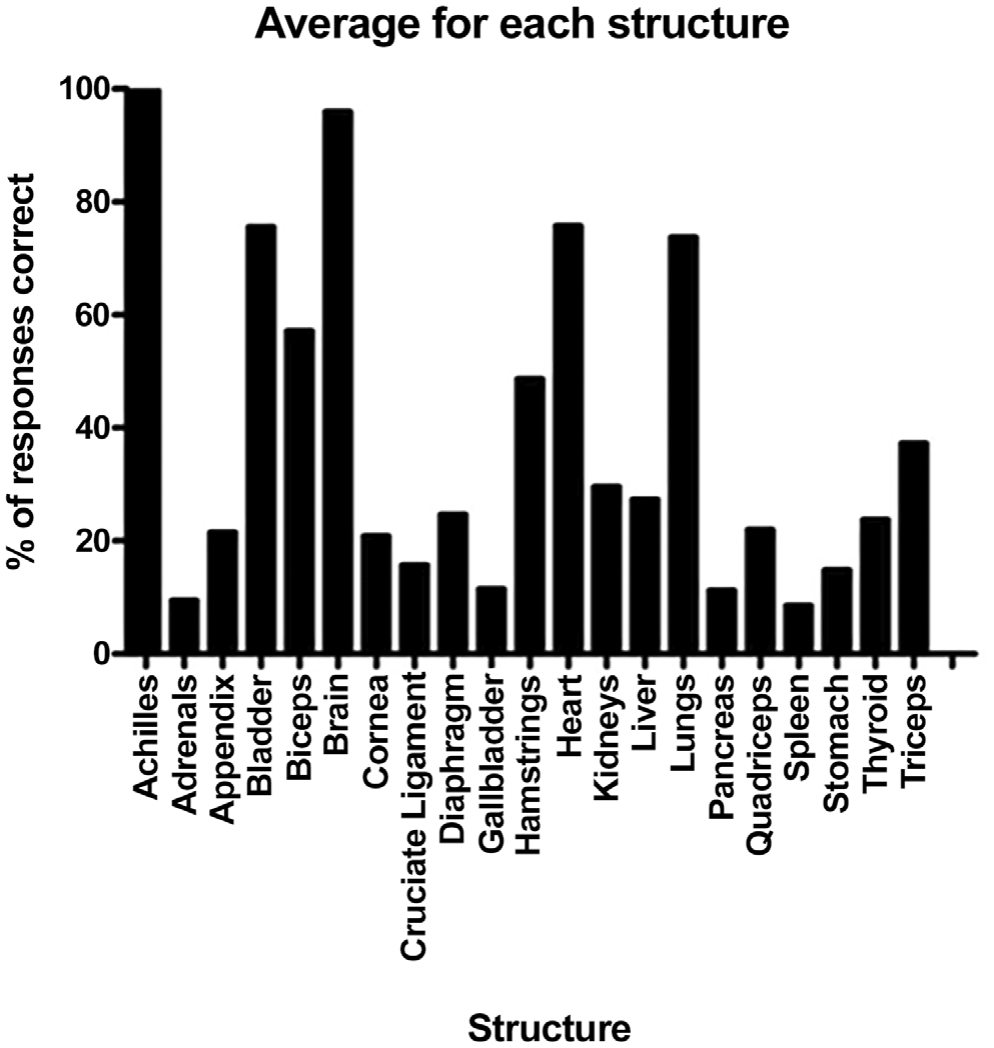
Overall scores showing the percentage of the 481 respondents who answered each structure correctly. Bar represents mean ± SEM.

**FIGURE 2:**
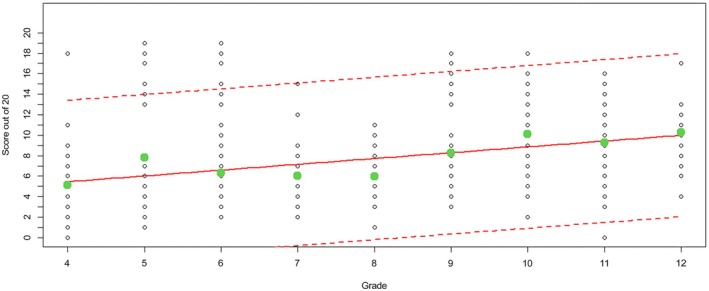
The score (out of 20) achieved by students for each grade (black circles), the regression line (red continuous line), and its 95% confidence intervals (red dashed lines) and the average number of correct answer for each grade (green circles).

The structures assessed as part of this study showed that across the 481 responses, the brain (96.3%), heart (75.8%), bladder (74.6%), and lungs (74.2%) performed best, with a number of musculoskeletal structures also showing strong results. Structures within the abdomen were most poorly answered, with the spleen (8.8%), adrenals (9.8%), gallbladder (11.9%), and pancreas (12.1%) performing most poorly across the cohort (Figure 1). The average score was 8.26 of 20 or 39% of structures correct; with the increase in grade, the average percentage of correct structures increased compared with the younger grades (Figure 2).

**FIGURE 3:**
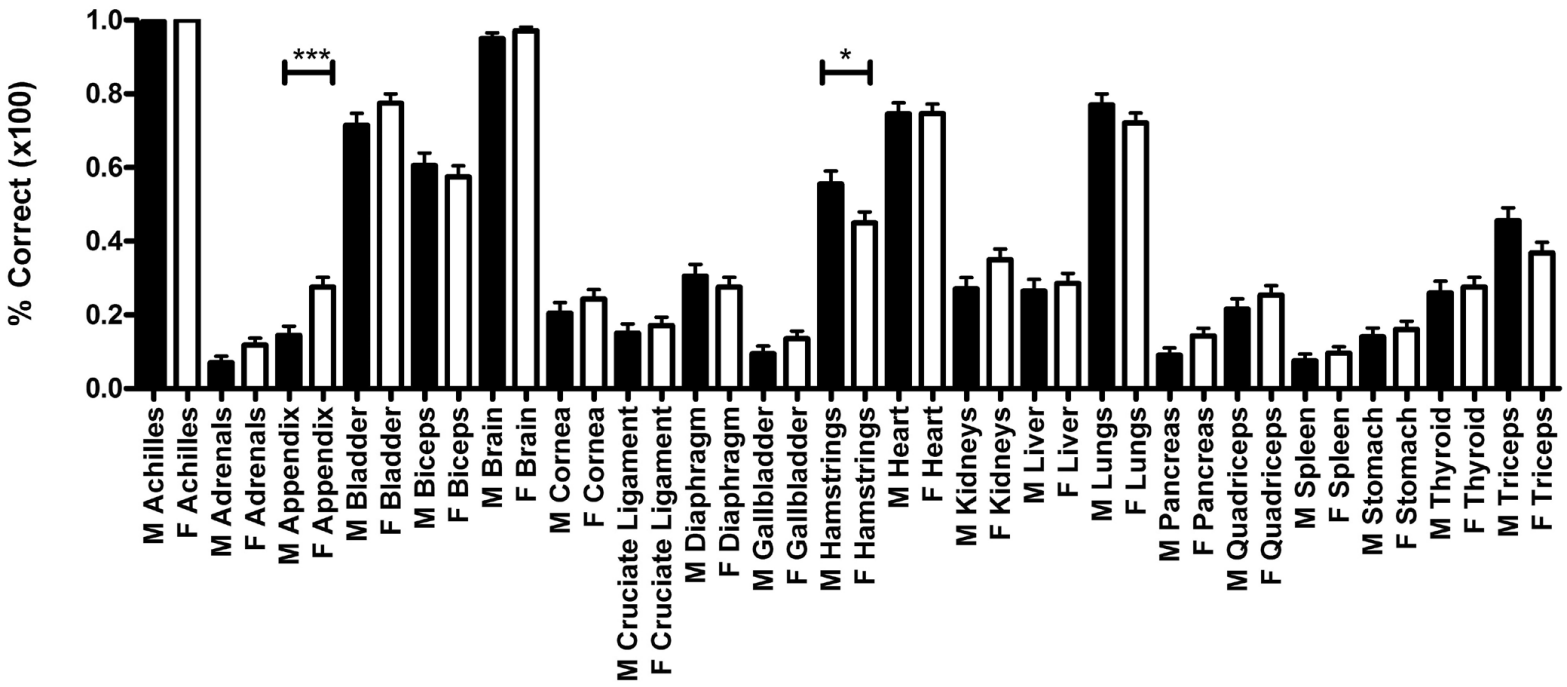
Overall scores showing the average correct score for each structure grouped into boys (black) and girls (white) for the 481 participants. Only the appendix; girls > boys (*p* ≤ 0.0001) and hamstrings; boys > girls (*p* ≤ 0.05) showed a significant difference. Girls performed better than boys in 15 of the structures assessed.

The responses also showed that the range of percentage correct answers decreased, suggesting a more consistent level of anatomical knowledge in relatively older boys and girls within this study (Figure 2). Girls demonstrated a stronger score in numerous structures compared with the boys in their respective grades (Figure 3).

Performance is associated with grade, with most grades above 4 improving in their performance when compared with grade 4 (reference category, Table 2). Exceptions are grades 7 and 8. Grade 10 has the largest performance compared with grade 4, with students in grade 10 doubling the performance of students in grade 4 when controlling for gender and school.

**TABLE 2:** Generalized linear binomial mixed model results.

Variable	Estimate odds ratio	*p*‐value
Intercept	0.24	<0.001
Grade 4	*Ref*	
Grade 5	1.53	<0.001
Grade 6	1.23	0.01
Grade 7	1.16	0.12
Grade 8	1.17	0.09
Grade 9	1.69	<0.001
Grade 10	2.03	<0.001
Grade 11	1.80	<0.001
Grade 12	1.84	<0.001
Female	*Ref*	
Male	1.03	0.38

Gender was not statistically associated with overall performance (*p*‐value above 0.05). Although overall performance was not statistically significant between boys and girls, there were two structures that showed significant differences. The first was where boys outperformed girls (hamstrings; 56% vs. 45%, respectively, *p* < 0.05) and the second was where girls outperformed boys (appendix; 28% vs. 15%, respectively, *p* < 0.001) (Figure 3), when not adjusting for the grade.

As the structures in our survey are seldom taught alone, usually as part of a system, we next examined the performance of each grade of students for each of the body systems as aligned with the curriculum and structures in our survey.

**FIGURE 4:**
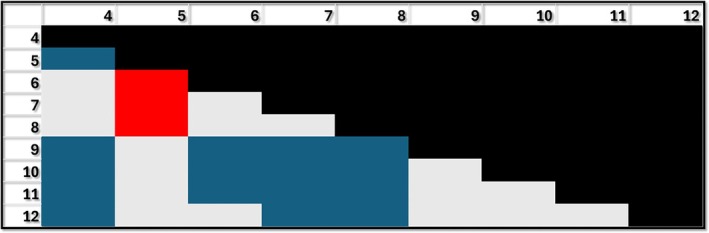
Performance for the endocrine system by grade. Blue: Grade shown in row performing better than the grade shown in column (e.g., second row and first column: Grade 5 performing better than grade 4); Red: Grade shown in row performing worse than the respective grade in column (e.g., 6 performing worse than 5); Gray: No difference between grades. The differences shown in blue and red are statistically significant *p* < 0.05.

For the endocrine system, grades 5, 9, 10, 11, and 12 performed significantly better than grade 4, with grades 6, 7, and 8 performing significantly worse than grade 5. Grades 9, 10, and 11 performed significantly better than 6, 7, and 8, while grades 10, 11, and 12 performed significantly better than grades 7 and 8. Although only grades 10 and 11 cover the endocrine systems, these grades, along with 9 and 12 significantly outperform all the earlier grades but not grade 5 (Figure 4).

**FIGURE 5:**
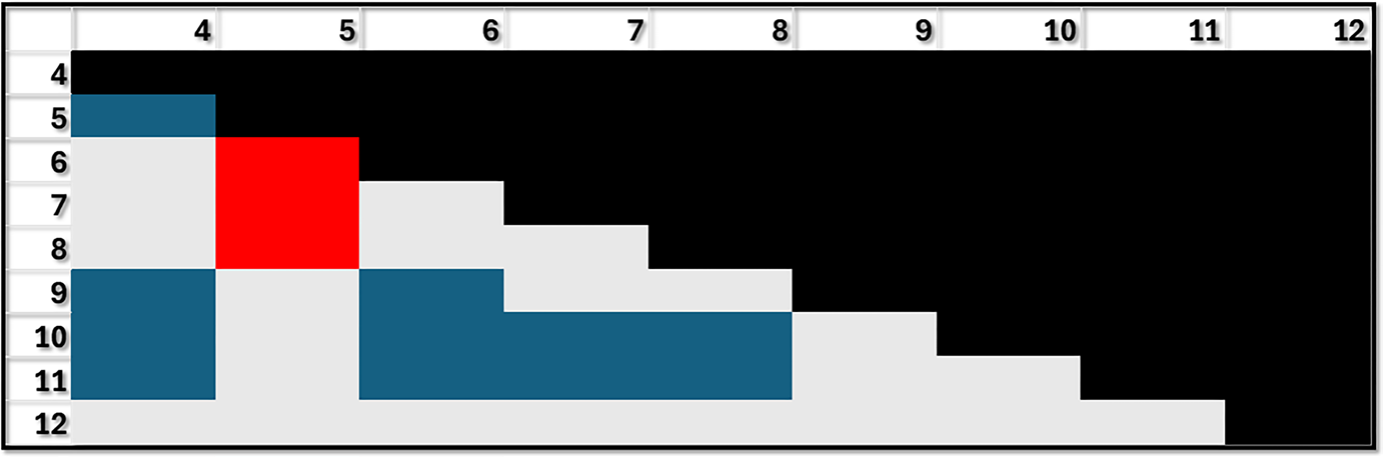
Performance for the digestive system by grade. Blue: Grade shown in row performing better than the grade shown in column (e.g., second row and first column: Grade 5 performing better than grade 4); Red: Grade shown in row performing worse than the respective grade in column (e.g., 6 performing worse than 5); Gray: No difference between grades. The differences shown in blue and red are statistically significant *p* ≤ 0.05.

Similar significant effects were found for the digestive system, although grade 12 was not different from any other grade. Grades 5, 9, 10, and 11 perform significantly better than grade 4. It is interesting to note that grades 6–8 performed significantly worse than grade 5. Grades 9–11 all performed better than grades 6 and grades 10 and 11 performed significantly better than grades 7 and 8. (Figure 5).

**FIGURE 6:**
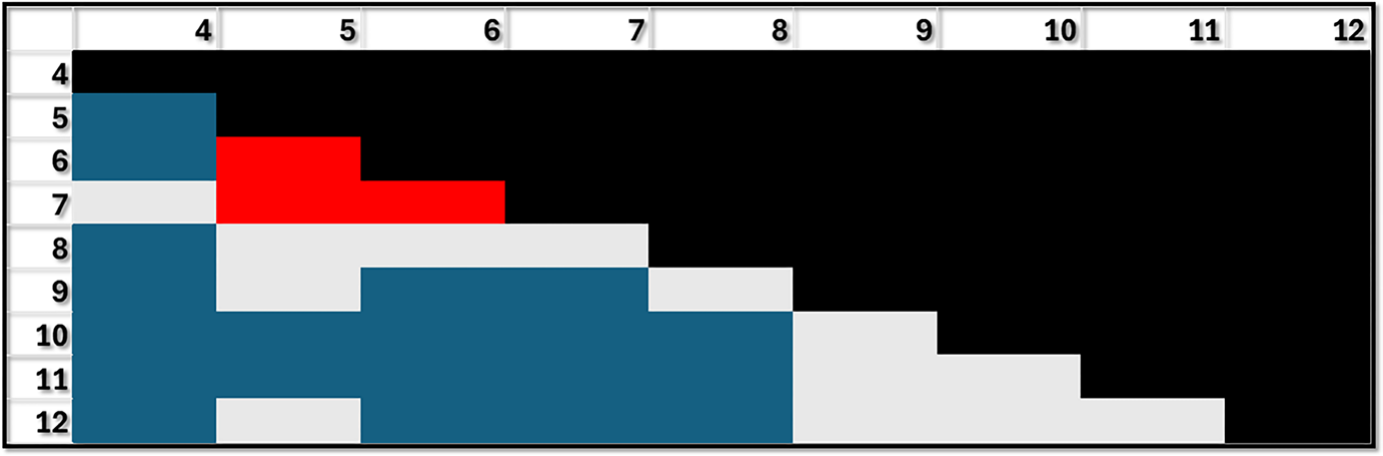
Performance for the muscular system by grade. Blue: Grade shown in row performing better than the grade shown in column (e.g., second row and first column: Grade 5 performing better than grade 4); Red: Grade shown in row performing worse than the respective grade in column (e.g., 6 performing worse than 5); Gray: No difference between grades. The differences shown in blue and red are statistically significant *p* ≤ 0.05.

For the muscular system, most of the grades performed significantly better than grade 4 with grade 7 as the only exception. Grades 6 and 7 performed significantly worse than grade 5, and grade 7 significantly worse than grade 6. These results are particularly concerning for grade 7, given that grades 5, 7, and 9 are the only three grades covering the muscular system (but grade 5 outperforms grades 4, 6, and 7). Grade 9 and above tended to significantly outperform all the previous grades with a few exceptions (grades 5 and 8) (Figure 6).

**FIGURE 7:**
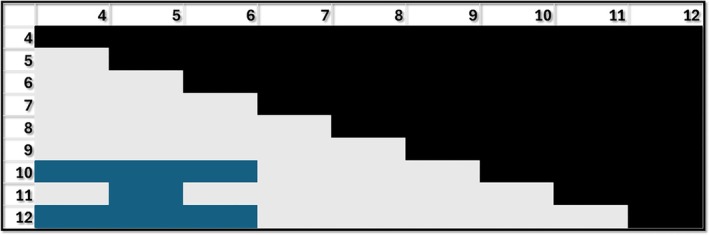
Performance for the respiratory system by grade. Blue: Grade shown in row performing better than the grade shown in column (e.g., grade 10 performing better than grade 4); Gray: No difference between grades. The differences shown in blue are statistically significant *p* ≤ 0.05.

Grades 10 and 12 performed significantly better than grades 4, 5, and 6 for the respiratory system, and grade 11 better than 5. No differences were found between grades 7 and 12 (Figure 7). This system showed the least number of significant differences between grades (Table 3).

**TABLE 3:** References to anatomy and/or human body systems in the school curricula in Namibia.^36^, ^37^, ^38^, ^39^

	Grade 4	Grade 5	Grade 6	Grade 7	Grade 8	Grade 9	Grades 10 and 11	Grade 12
	(9–10)	(10–11)	(11–12)	(12–13)	(13–14)	(14–15)	(15–16/16–17)	(17–18)
Personal health	Y		Y					
Dental	Y							
Respiratory	Y		Y		Y	Y		Y
Excretory	Y			Y		Y	Y	
Development	Y		Y	Y				
Circulation		Y	Y	Y		Y	Y	Y
Reproduction		Y	Y	Y		Y	Y	
Digestion		Y		Y	Y		Y	
Skeleton		Y		Y		Y		
Nervous		Y		Y		Y	Y	
Muscular		Y		Y		Y		
Immune						Y		Y
Endocrine							Y	

*Note*: Grade listed in each column with child ages listed in bracket. White filled boxes denote primary school grades, gray filled boxes denote secondary grades. Grades 10 and 11 are combined as they form part of a 2‐year syllabus that was implemented nationally in 2019, the body systems covered in this are stipulated as part of this syllabus and are not divided into grade 10 or 11 as the relevant year of teaching.

**FIGURE 8:**
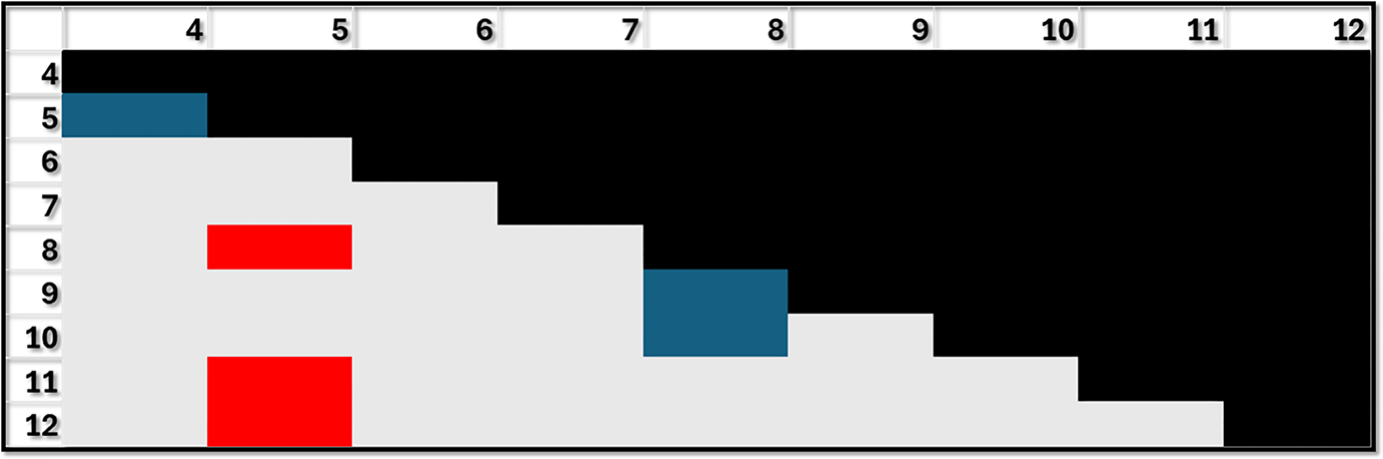
Performance for the immune system by grade. Blue: Grade shown in row performing better than the grade shown in column (e.g., second row and first column: Grade 5 performing better than grade 4); Red: Grade shown in row performing worse than the respective grade in column (e.g., 8 performing worse than 5); Gray: No difference between grades. The differences shown in blue and red are statistically significant *p* ≤ 0.05.

In the immune system, grade 5 performed better than grade 4, and 9 and 10 performed better than grade 8. It is interesting to note that grades 8, 11, and 12 performed worse than grade 5. Grade 12 is the only grade with the immune system in its curriculum. As in most of the systems, grade 5 outperforms or is not different from higher grades (Figure 8).

No statistically significant difference in performance, based on grade and gender, was found for excretory, nervous, and circulatory systems.

Finally, only for the muscular system, boys performed better than girls in all grades, while no difference was found for the other systems. In all body systems, grades 9 to 12 do not show statistically significant differences in performance between them.

Our model also demonstrated that overall, students performed significantly better in their knowledge of the following systems as the grade increased, compared with the lower grades, in order of best to worst: muscular, endocrine, respiratory, digestive, and nervous, these systems showed the greatest improvement in performance. The data was inconclusive in demonstrating improved performance in knowledge for excretory, circulatory, and immune systems. In all systems, higher grades outperformed lower.

**FIGURE 9:**
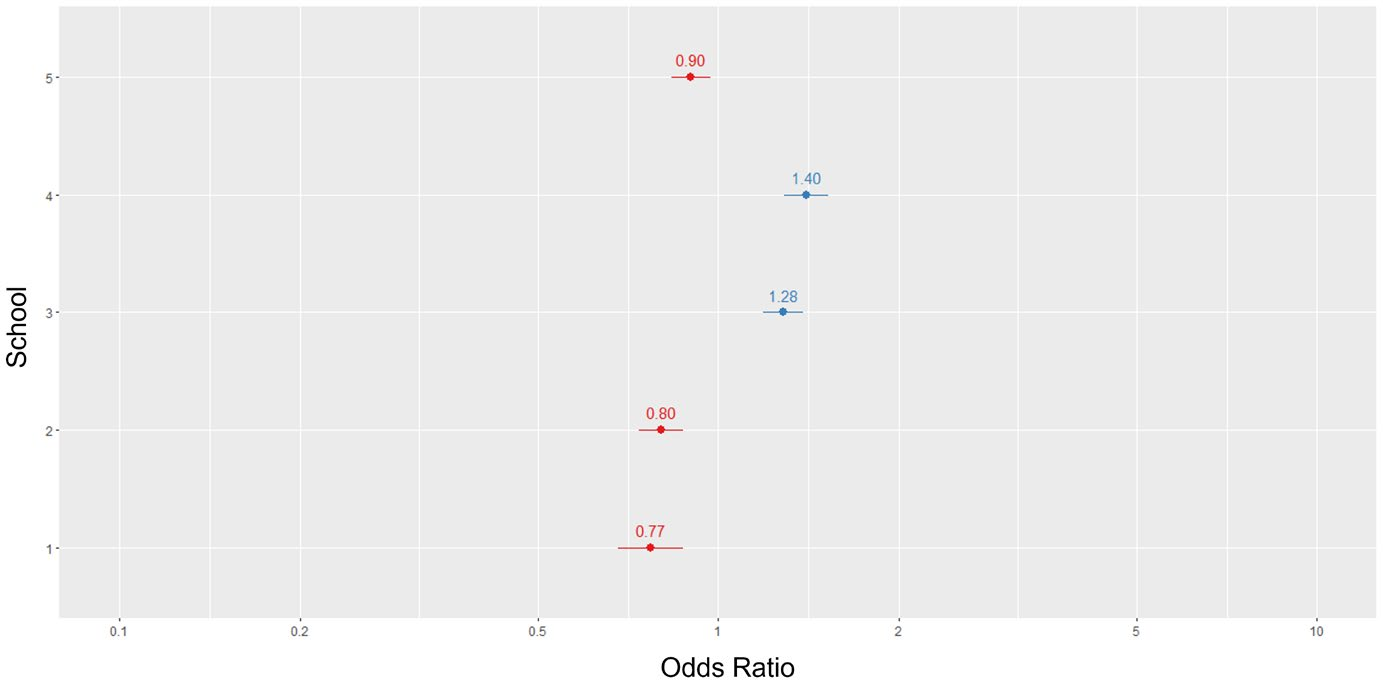
A forest plot demonstrating the relative performance of schools compared with the benchmark (1) the average performance of all students (regardless of school) after correcting for grades and gender.

Two schools, scored significantly above the benchmark, the average performance of all students (regardless of school) after correcting for grades and gender. One scored notably higher than three of the four other schools and better than one school number: 63%, 60%, 50%, and 11% higher, respectively. Three of the schools, scored significantly worse than the benchmark (Figure 9).

## DISCUSSION

This study represents the first assessment of anatomical knowledge of school children. The 481 responses represent the largest assessment of public knowledge of anatomical structures in recent years, with the overall mean of the cohort being 8.26 of 20 (39%) of structures correct. The correct average response rates of the current study were higher than the 6.5 of 20 reported in Hong Kong, but less than our previous study in the UK which gave an average of 11 of 20 correct responses.^3^, ^6^ Furthermore, it represents the first anatomical knowledge assessment of a cohort of Namibian and African individuals, with previous recent studies demonstrating the presence of some juveniles in their larger cohorts in the UK and Hong Kong. In the UK study, the average age was 36.5 years; in the Hong Kong study, the average age is not known, but it is assumed from the data available to be above that of our study. Multiple studies have shown that there is increasing anatomical knowledge of children as they age,^40^, ^41^, ^42^ but it is clear from some of the other studies that this knowledge declines in early adult years before increasing again around or beyond middle age.^3^

In this study, there is a clear increase in anatomical knowledge from the start of the school grades through to the end, grades 4 to 12, respectively, this is consistent with child performance in other studies.^3^, ^6^ This increase in knowledge is statistically significant, although not in the final years of primary school—grades 5 and 6. In the Namibian curriculum, across these initial 3 grades; grades 4 and 5 see the first exposure to each of the body systems and learning of new knowledge. It is not until grade 6 that the spiraling exposure of some of these systems (development, circulatory, and human reproduction) appears again. Although exposure to anatomy‐related material in other areas of life cannot be ruled out and raises a question that warrants further investigation where children are taught about or acquire knowledge about human anatomy. There are several obvious sources: at school, at home, through TV, online media, or literature (both fiction and non‐fiction) and through their peers.

Studies dating back to 1953 have advocated for increased human anatomy instruction in schools. Research has shown a significant association between higher anatomical knowledge in children and their fathers' educational background. Specifically, children whose fathers graduated college scored significantly higher.^43^ Furthermore, younger children with fathers who had higher levels of education also demonstrated greater anatomical knowledge when their parents' education and occupations were analyzed against their performance.^44^, ^45^, ^46^, ^47^

Earlier studies have used a variety of methods to determine the anatomical knowledge of school students, the vast majority and as seen in this study, have asked students to draw what they think is within the body when provided with a blank outline.^48^, ^49^, ^50^ Other studies have used a more open assessment, relying on participants' ability to position organs on a toy doll. It is suggested that utilization of a blank outline gives a clearer representation of an individual's true level of anatomical knowledge over multiple‐choice options, as multiple‐choice testing could trigger partially remembered knowledge.^28^ Where most of the public including children encounter anatomical knowledge, such as in a healthcare setting, the availability of information to trigger partially remembered information may not be a terrible thing. Within the context of this study, the methodology represents a solid open assessment of their underlying knowledge of topographical anatomy.

In this study, the increase in performance from the younger grades to the older grades for almost all structures fits with the understanding that as children age, they increase their knowledge about the body. Their progression through the developmental stages detailed in the introduction has been shown to play a role in how they will describe and understand anatomy.^51^, ^52^

Some of the results in this study align with these findings, the largest gain in knowledge is seen across musculoskeletal structures, as children hit the formal operational stage, they are encountering puberty, where hormones begin to exert their influence on the body, particularly the musculoskeletal system.^53^, ^54^ Although they cannot see the changes beneath the skin, the significant effect hormones are having effect on the musculoskeletal system, increasing size and mass of muscles and bones, may be evident visually on themselves or when observing peers, these changes also increase forces that are responsible for foundational motor skills such as running and jumping. These changes might account for the stronger performance on this anatomical system compared with some of the others which are much harder to visualize or see the changes in.

The performance of the students in this study shows a progressive increase in anatomical knowledge. The increasing knowledge across many of the systems, as the school grade increases suggests that students are acquiring and retaining knowledge as they progress through their school years. Previous research has suggested that around the age of 10 years is when children transition from describing their digestive structures as “the tummy” to using proper terminology such as the stomach.^55^ The significantly improved performance of students from grade 9 onwards suggests that correct anatomical structures and their relationship inside the abdomen are well understood compared with earlier years.

With the strong performance in their formative years, there are questions about what happens to this knowledge in some of the years beyond formal education, as a decline in knowledge has been shown in later decades.^3^ ^,^ ^56^, ^57^, ^58^ This knowledge is valuable as we age and begin to see health problems arise; lower levels of health and body knowledge correlate with difficulty or delay in accessing appropriate care, which can translate to worse outcomes.^59^ The importance of the influence of education is well established and leads to better overall health and a longer lifespan. The opportunity for this kind of knowledge to have a positive impact is significant. In Namibia, the top 3 leading causes of death are communicable diseases and account for 43% of deaths in the country in 2019 (before Covid‐19 became a significant contributor to the number of deaths from communicable diseases across the globe).

Knowledge about specific organs, structures, and systems is important and varies from country to country in terms of the link(s) to the most common causes of morbidity and mortality.

Overall performance on each structure showed that there were very limited significant differences between girls and boys, although girls were better at answering 15 out of the structures; all but one of those that boys were better at answering were muscles, with lungs being the non‐muscular structure. In our study, as with previous studies, it has been shown that boys are better at answering the location of muscles, but not viscera compared with girls.^3^

It is interesting to note that girls were better at answering three‐quarters of the structures, with the appendix being the only one that was answered significantly better. The reasons for this are unknown but it may be down to the girls' exposure to and experiences of the menstrual cycle which could assist in gaining knowledge about the menstrual cycle, associated structures and potential pains associated with this bodily process. This may make girls more aware of other types of pain that are associated with abdominal organs and their location, such as the appendix, but there is limited data supporting this. Girls have been shown to be better at answering viscera but other evidence suggests that knowledge of genitalia and structures involved in the menstrual cycle in girls and women has room for improved understanding, even in individuals who are suffering from conditions affecting these structures.^3^, ^8^, ^60^, ^61^, ^62^

The authors recognize that there is a limited significant difference in the results when assessing the performance between girls and boys in this study. Although not significant, the results are still relevant; in this study, we argue that this lack of significance is fundamentally important. In a country and region where historically, girls are often absent and overlooked in education for a variety of reasons, this cohort at a variety of schools across the country demonstrates what can be achieved when girls have equal access to educational provision at all ages within the school system.

It is recognized that boys and girls in Namibia have similar performance at primary school, but this disappears in further years of education. In terms of anatomical learning, our study shows that there is similar performance in later years when girls are given similar access to education compared with boys. This could be an example of what is achievable when policies such as those being enacted in Namibia are doing to support girls in education; further work in a public educational setting would be a useful comparator. The vast geographical size and varied population location and demographics mean that challenges still persist and time to overcome these may still be a significant time away for some areas of the country, but examples of positive performance for girls in educational settings can be a showcase for what is attainable and make a case for further government support or reinforcing those actions already taken.

Many of these challenges that girls face, such as expectations to take on household responsibilities such as caring for siblings and elderly parents have a negative impact on the time that they may get in education compared with their boy counterparts. Similarly, in some of these areas, early marriage, sexually transmitted diseases, and potential pregnancies remain key barriers to girls' educational attendance and progress. Where education and safe access to it for girls exist, this may help parents who are reluctant to send their daughters to school, particularly secondary school due to safety concerns during these journeys in rural or remote areas overcome many of these fears and barriers.^63^, ^64^, ^65^, ^66^, ^67^

Economic challenges also disproportionately affect girls in sub‐Saharan countries such as Namibia. Primary education across Namibia is free, but this does not include costs associated with schooling such as transportation, where available, cost of uniforms or any reading or writing materials that may be required. This often results in boys being prioritized for attending school over their sisters. This disproportionately affects girls, especially in poorer families, as they may be required to work at home providing care or helping with family existence such as farming. Any of these situations will result in girls having less access to educational time and the expectation that they would perform more poorly compared with their boy counterparts.^68^, ^69^, ^70^

Our study, which included a greater proportion of girls and young women (58% of respondents), is potentially unexpected to see them matching and outperforming their boy classmates considering the historical inequity. In sub‐Saharan countries, as many as 41% of school‐aged adolescent women have reported missing school due to menstruation and/or symptoms such as pain, reporting more missed school calendar days than boys.^71^, ^72^, ^73^ Similar issues affect girls in these age groups as they are often responsible for the carer role in their families, as well as slowing or terminating their educational journey due to pregnancy, which in some sub‐Saharan countries can be over 40,%; both are further reasons why they often demonstrate an increased absence rate from school.^68^, ^71^, ^72^, ^74^ Namibia continues to face challenges in relation to teenage pregnancy, with 66 teenage births per 1000 girls aged 15–19 in 2023; this is above the global average of 44 and statistics show that in 2023.^75^ In the 3 years between 2018 and 2021, Namibia documented 56,300 teenage pregnancies, which is almost 20,000 more than the number of teenagers who qualified for university entry (37,480).^76^ The huge geographical area and diverse population across city, suburban, and rural communities may contribute to this.

Although it is outside the scope of this article, it is important to note that change may be on the horizon in Namibia as government policies such as the National Gender Policy, aim to reduce gender disparities across all sectors, not just education. In countries such as Namibia, where there is heavy investment in education, this serves as evidence that health knowledge is being instilled at an early age and there is an opportunity for it to add self and societal value going forward.^77^, ^78^ In other developed and developing countries, this knowledge has been shown to be lacking in later decades which has the potential to increase poor outcomes in healthcare access and diagnoses.^69^, ^70^ This is particularly important in Namibia which has a particularly poor life expectancy, one of the shortest in the world at 60.4 years of age, which is well below the average of similar economically developed countries.^79^ The performance of students in this study shows the promising understanding that Namibian children have about their anatomy. This has potential for the future to help them build on their own health literacy, helping themselves and their family members to be better able to assimilate themselves with how they understand and access information relating to their health.^80^ This is particularly important in light of the life expectancy in Namibia and some of the health challenges the population faces.^81^, ^82^

One point of potential bias or skewing is that all the schools that participated in this study are private schools within Namibia; private schools accounted for 6% of the 819,749 pupil enrolments across the country in 2022.^19^ They must follow the national curriculum unless special dispensation is obtained from the government to adapt. Namibia invests heavily in its education with 9.4% of its GDP being spent on education.^30^ Private schools in sub‐Saharan Africa perform much better than state‐funded schools, and their advantage is positive and moderately large.^83^, ^84^ The authors also note that there is variation in the performance of the different schools that participated; there are several reasons why this might be more than just the curriculum itself. For instance, studies elsewhere have demonstrated a statistically significant relationship in learners' scores for biology when comparing the International General Certificate of Secondary Education and the International Baccalaureate Diploma Programme.^85^ However, subject‐specific comparative data do not exist for Namibia. Additional considerations that might impact learners' performance include the extent of curriculum implementation, teacher experience and subject knowledge, available teaching resources, timepoint of involvement in our study compared with relevant material delivery through the curricula for each institution and continuous assessment practices.^85^

Further work looking at the performance of a similar cohort in the public school system would enable a valuable comparison between the two educational strands and give a more complete indication of the anatomical knowledge in the majority of Namibian school children.

## CONCLUSIONS

Namibian private school children have shown a strong and progressing knowledge of anatomy across ages 9–18. Children typically demonstrate a keen knowledge of body parts, although not known by their anatomical names, as toddlers.^41^ This study also shows what can happen to girls' education and the knowledge they can achieve when given access to equal educational time and resources compared with boys of the same age. In terms of anatomical structures and body systems knowledge in the later years of secondary school, performance plateaus, and peaks around age 15.

## AUTHOR CONTRIBUTIONS

**Adam M. Taylor:** Conceptualization; writing – review and editing; project administration; formal analysis; writing – original draft; methodology; validation; data curation. **Lojandrie Kirsten:** Writing – original draft; investigation; methodology; formal analysis; project administration; validation. **Luigi Sedda:** Writing – review and editing; formal analysis; validation; writing – original draft; project administration; methodology; conceptualization. **Quenton Wessels:** Writing – review and editing; formal analysis; project administration; supervision; data curation; methodology; validation; writing – original draft; conceptualization; investigation.

## ETHICS STATEMENT

The study was approved by the Namibian Ministry of Health and Social Services (reference #: LK2022).
